# Biofilm Formation by *Histoplasma capsulatum* in Different Culture Media and Oxygen Atmospheres

**DOI:** 10.3389/fmicb.2020.01455

**Published:** 2020-07-10

**Authors:** Larissa Naiara Carvalho Gonçalves, Caroline Barcelos Costa-Orlandi, Níura Madalena Bila, Carolina Orlando Vaso, Rosângela Aparecida Moraes Da Silva, Maria José Soares Mendes-Giannini, Maria Lucia Taylor, Ana Marisa Fusco-Almeida

**Affiliations:** ^1^School of Pharmaceutical Sciences, Department of Clinical Analysis, Universidade Estadual Paulista (UNESP), Araraquara, Brazil; ^2^School of Veterinary, Department of Para Clinic, Universidade Eduardo Mondlane, Maputo, Mozambique; ^3^School of Medicine, Department of Microbiology and Parasitology, Universidad Nacional Autónoma de México, Mexico City, Mexico

**Keywords:** *Histoplasma capsulatum*, biofilms, culture media, oxygen atmospheres, virulence factors

## Abstract

*Histoplasma capsulatum* is a dimorphic fungus that causes an important systemic mycosis called histoplasmosis. It is an infectious disease with high prevalence and morbidity that affects the general population. Recently, the ability of these fungi to form biofilms, a phenotype that can induce resistance and enhance virulence, has been described. Despite some efforts, data regarding the impact of nutrients and culture media that affect the *H. capsulatum* biofilm development *in vitro* are not yet available. This work aimed to study *H. capsulatum* biofilms, by checking the influence of different culture media and oxygen atmospheres in the development of these communities. The biofilm formation by two strains (EH-315 and G186A) was characterized under different culture media: [Brain and Heart Infusion (BHI), Roswell Park Memorial Institute (RPMI) with 2% glucose, Dulbecco’s Modified Eagle’s Medium (DMEM) supplemented with 10% fetal bovine serum and nutrient medium HAM-F12 (HAM-F12) supplemented with glucose (18.2 g/L), glutamic acid (1 g/L), HEPES (6 g/L) and L-cysteine (8.4 mg/L)] and oxygen atmospheres (aerobiosis and microaerophilia), using the XTT reduction assay to quantify metabolic activities, crystal violet staining for biomass, safranin staining for the quantification of polysaccharide material and scanning electron microscopy (SEM) for the observation of topographies. Results indicated that although all culture mediums have stimulated the maturation of the communities, HAM-F12 provided the best development of biomass and polysaccharide material when compared to others. Regarding the oxygen atmospheres, both stimulated an excellent development of the communities, however in low oxygen conditions an exuberant amount of extracellular matrix was observed when compared to biofilms formed in aerobiosis, mainly in the HAM-F12 media. SEM images showed yeasts embedded by an extracellular matrix in several points, corroborating the colorimetric assays. However, biofilms formed in BHI, RPMI, and DMEM significantly induced yeast to hyphae reversal, requiring further investigation. The results obtained so far contribute to *in vitro* study of biofilms formed by these fungi and show that nutrition promoted by different media modifies the development of these communities. These data represent advances in the field of biofilms and contribute to future studies that can prove the role of these communities in the fungi-host interaction.

## Introduction

Histoplasmosis is a systemic mycosis widely distributed worldwide with high prevalence in America (Mittal et al., 2019). Most cases are reported in Ohio and Mississippi River valleys in the United States, Mexico, Brazil, and some regions of Guyana (Guyana Shield). The etiologic agent of histoplasmosis is *Histoplasma capsulatum* and, recently, Sepúlveda et al. (2017) reported that this species is composed of at least four cryptic species that differ in virulence and genetics (*H. capsulatum, Histoplasma suramericanum, Histoplasma ohiense e Histoplasma mississippiense*). It is considered the most prevalent agent in respiratory infections caused by fungi registered in recent years (Hage et al., 2015). The annual incidence of histoplasmosis is around 500,000 cases. In HIV/AIDS patients, infection rates range from 2 to 25% in endemic areas, causing approximately 60% of death (Adenis et al., 2014a; Bongomin et al., 2017; Limper et al., 2017). The scientific advancement of recent years has helped to clarify that the formation of biofilm represents the common way that microorganisms are found in nature (Sardi et al., 2015). Biofilms are very important for public health, as they are related to high levels of resistance to antimicrobial agents (Nett et al., 2014; Soll and Daniels, 2016). Approaches to fungal biofilms are widely discussed in the literature. Several fungi have demonstrated the ability to colonize surfaces and form biofilms, including: *Candida* spp. (Hawser and Douglas, 1994), *Cryptococcus neoformans* (Martinez and Casadevall, 2005), *Saccharomyces cerevisiae* (Verstrepen and Klis, 2006), *Aspergillus* spp. (Seidler et al., 2008), *Paracoccidioides brasiliensis* (Sardi et al., 2015), *Trichophyton rubrum* and *Trichophyton mentagrophytes* (Costa-Orlandi et al., 2014) and *H. capsulatum* (Pitangui et al., 2012; Brilhante et al., 2015).

Fungal biofilms are sessile communities of microorganisms, which cells adhere to each other and to biotic or abiotic substrate, usually surrounded by an extracellular matrix of polysaccharides (Costa-Orlandi et al., 2017). Biofilm cells have structural, functional, and genetic differences in relation to planktonic cells (Kuhn and Ghannoum, 2004; Mowat et al., 2007; Ramage et al., 2009; Pitangui et al., 2012, 2016). The biofilm morphology can be influenced by the quality and quantity of available nutrients, such as the composition of the culture medium (Kucharíkova et al., 2011; Lee et al., 2013; Serrano-Fujarte et al., 2015) and the oxygen concentration (Stichternoth and Ernst, 2009; Bonhomme et al., 2011).

The biofilms structural characterization study combines colorimetric and microscopic techniques. These tests evaluate from the amount of metabolically active cells in the community, the amount of biomass and polysaccharide material produced, in addition to the topography, density, and morphology of the biofilm (Costa-Orlandi et al., 2017). There are few studies in the literature related to the biofilm’s formation by these fungi (Pitangui et al., 2012; Brilhante et al., 2015). These studies must be deepened in order to find an *in vitro* condition that better provides the study of these communities *in vivo*. Therefore, this work aims to verify the influence of culture media and oxygen atmospheres in the biofilm formation by different strains of *H. capsulatum*, in order to discover the best conditions for the study of these biofilms *in vitro* and enhance the understanding of the possible formation of these communities *in vivo*.

## Materials and Methods

### Microorganisms and Growth Conditions

Two strains of *H. capsulatum* (EH-315 and G186A/ATCC 26029) were used for all experimental tests. The EH-315 strain was recovered from the intestine of an infected bat of the Mormoops species, captured in a cave in the state of Guerrero in Mexico. This strain was deposited in the Culture Collection of the Fungus Immunology Laboratory of the Department of Microbiology and Parasitology of Universidad Nacional Autónoma de México (UNAM)^1^. This strain was registered in the World Federation for Culture Collection database under the number LIH-UNAM WDCM817. The G186A strain (ATCC 26029), Panamá, is a wild type strain provided by Dr. Roseli Zancopé Oliveira of the Oswaldo Cruz Foundation (Fiocruz) of Rio de Janeiro. The yeast phase of each strain was maintained in Brain and Heart Infusion (BHI) agar (Difco Laboratories) (Goughenour et al., 2015).

### Biofilm Formation by *H. capsulatum* in Different Nutrient Conditions and Oxygen Atmospheres

The strains were removed from the maintenance medium and subsequently subcultured in the Ham’s F-12 Nutrient Mixture medium (HAM-F12) (Gibco) supplemented with 1,8% glucose (Synth), glutamic acid (Synth), HEPES (Sigma) and 0.1% L-Cysteine (Synth) at 37°C, for 96 h and with shaking at 150 rpm. The contents were dispensed in 50 mL conical tubes (Corning), which were centrifuged at 5000 rpm, at 30°C for 10 min. The supernatant containing the medium was removed and the pellets were washed three times with sterile phosphate buffered saline (PBS). After washing, the cells were resuspended in sterile PBS and the cell viability was checked in a hemocytometer using the Trypan blue (Gibco) in a 1:1 ratio. At least 90% viability certified, viable cells were counted, and fungal suspensions were prepared in sterile PBS at 5 × 10^6^ céls/mL. Two hundred microliters were added to the 96-well plates (Kasvi), which were incubated for 12 h for pre-adhesion period (pre-established time) in two conditions (aerobiosis, at 37°C with shaking at 150 rpm and in microaerophilia atmosphere formed in candle jar (Mandal et al., 2014 and Carr et al., 2015), at 37°C, without shaking). After pre-adhesion, supernatants were removed, and cells were washed three times with sterile PBS to remove non-adherent cells. Finally, 200 μL of different culture media [(Brain–Heart Infusion (BHI-broth) (Kasvi) and supplemented with 0.1% L-cysteine (Synth) and 1% glucose (Synth), Ham’s F-12 Nutrient Mixture medium (HAM-F12) (Gibco) supplemented with (18.2 g/L) glucose (Synth), (1 g/L) glutamic acid (Synth), (6 g/L) HEPES (Sigma) and (8.4 mg/L) L-cysteine (Synth), Dulbecco’s modified Eagle’s medium (DMEM) (Sigma-Aldrich) supplemented with 10% heat-inactivated fetal calf serum (Gibco) and Roswell Park Memorial Institute (RPMI) 1640 medium, with L-glutamine, without sodium bicarbonate (Gibco) and buffered with MOPS (Sigma-Aldrich), plus 2% glucose (Synth)], were added to the wells, followed again by incubation at 37°C in the same conditions mentioned above for 24–168 h (Pitangui et al., 2012; Costa-Orlandi et al., 2014).

### Determination of the Biofilm Metabolic Activity by the XTT Reduction Assay

The metabolic activities of the biofilms formed in the different conditions were verified by the XTT [2,3-bis- (2-methoxy-4-nitro-5-sulfophenyl) -5- (carbonyl (phenylamino)]-2H-tetrazolium hydroxide] reduction assay. Biofilms were formed in 96-well plates. After 24, 48, 72, 96, 120, 144, and 168 h of incubation, biofilm supernatants were removed, and cells were washed with sterile PBS to remove excess culture medium and non-adherent cells. Solutions of XTT (Thermo Fisher Scientific) (1 mg of salt / mL of PBS) and menadione (Sigma-Aldrich) (1 mM) in sterile PBS were prepared. Fifty microliters of XTT solution + 4 μL of menadione solution were added to the wells of the plates, which were subsequently incubated at 37°C for 3 h. After the incubation period, the optical densities of the communities (which are directly predictive of metabolic activity) were read on a Biotek Epoch^TM^ 2 spectrophotometer, at a wavelength of 490 nm (Martinez and Casadevall, 2007; Pitangui et al., 2012; Costa-Orlandi et al., 2014).

### Quantification of Biomass by Crystal Violet Staining

Biofilms were formed according to the conditions previously established. After incubation, the culture media were removed and 200 μL of methanol (Vetec) was added to the wells for 15 min. The supernatant was aspirated, and the plates were dried at room temperature. Then, 200 μL of 0.1% crystal violet (Dinamica) solution was added to each well for 20 min. After this period, the crystal violet was discarded, the wells were washed with sterile distilled water until the excess coloration was removed and the biofilms were bleached by the addition of 200 μL of 33% acetic acid (Synth). Finally, the plates were read in a spectrophotometer (Biotek Epoch^TM^ 2 Microplate Spectrophotometer), at a wavelength of 570 nm (Marcos-Zambrano et al., 2014).

### Quantification of Polysaccharide Material by Safranin Staining

The polysaccharide material of the biofilms was quantified by staining with safranin, as described by Seidler et al. (2008) and Costa-Orlandi et al. (2014). After the established times, the culture media were removed and 50 μL of 1% safranin solution was added to the wells for 5 min. The supernatants were subsequently removed, and the plates were washed with sterile 0.85% saline solution, until complete removal of the excess dye. Finally, the plates were read on a spectrophotometer (Epoch^TM^ 2 Microplate Spectrophotometer), at a wavelength of 492 nm.

### Scanning Electron Microscopy

For scanning electron microscopy (SEM), the biofilms were formed in 24-well plates (Kasvi) and samples were processed as described by Martinez and Fries (2010) and Costa-Orlandi et al. (2014), with minor modifications. After maturation, the biofilms were washed three times with sterile PBS to remove non-adherent cells. The communities were subsequently fixed with the addition of 800 μL of 2.5% glutaraldehyde solution (Sigma-Aldrich) and the plates were incubated under refrigeration at 4°C for 24 h. After this period, three washes were performed with 0.85% sterile saline solution. The samples were subsequently dehydrated with increasing concentrations of ethanol (Vetec) (25–100%) and were dried at room temperature. The bottoms of the plates were cut with a scalpel and, before analysis with a scanning electron microscope, the samples were mounted in aluminum cylinders with silver and placed in a high vacuum evaporator for the gold coating. The biofilms topographic characteristics were analyzed using a scanning electron microscope (Jeol 6610LV scanning JSM- at the School of Dentistry of UNESP at Araraquara, SP, Brazil).

### Statistical Analysis

The data were submitted to statistical analysis using the *t*-test or analysis of variance with Bonferroni’s *post test*, using the GraphPad Prism 5.0 software. *p*-values less than 0.05 were considered statistically significant (^∗^*p* ≤ 0.05, ^∗∗^*p* ≤ 0.01, and ^∗∗∗^*p* ≤ 0.0001). All experiments were performed in triplicate and with three independent experiments.

## Results

### Determination of the Metabolic Activity of Biofilms by the XTT Reduction Assay

**FIGURE 1:**
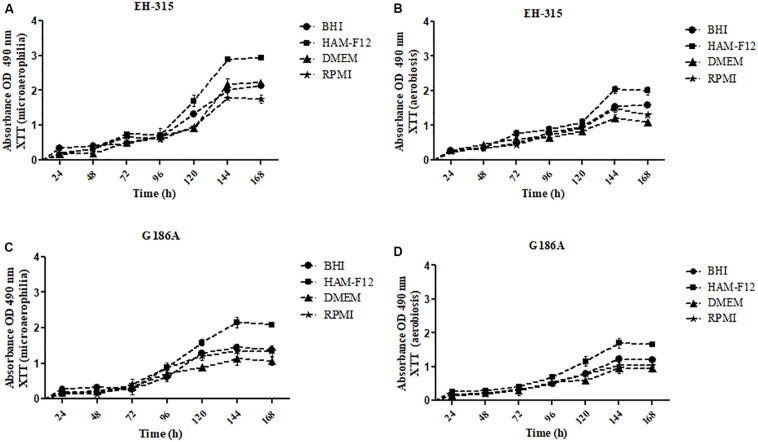
Kinetics of metabolic activities of biofilms formed by different strains of *H. capsulatum* **(A,B)** EH-315 and **(C,D)** G186A in microaerophilia **(A–C)** and aerobiosis **(B–D)** conditions.

The kinetics of the metabolic activities of biofilms formed by strains EH-315 and G186A are shown in Figure 1. The results indicated a slight increase in metabolic activities up to 96 h of incubation for both strains and in most conditions tested [microaerophilia (Figures 1A,C) and aerobiosis (Figures 1B,D)] and all culture media. From that time-point, there was a significant increase in metabolic activities, with exponential growth up to 144 h followed by a trend of stabilization and formation of a *plateau*. Thus, the 144 h period was considered the ideal time for biofilm maturation in all culture media and on both oxygen tensions.

**FIGURE 2:**
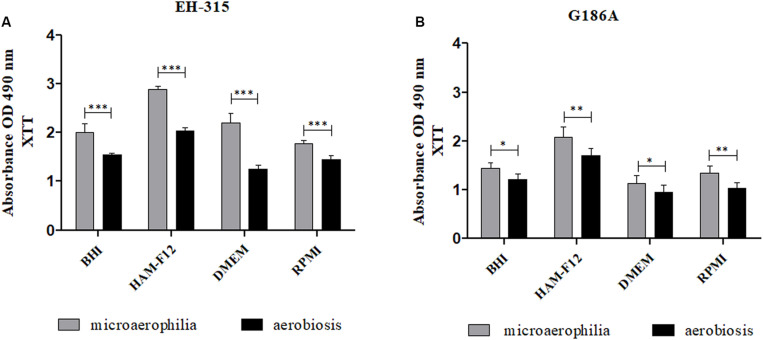
Comparison between the metabolic activities of biofilms formed by the strains *H. capsulatum* EH-315 **(A)** and G186A **(B)** in different culture media and under different oxygen atmospheres. *p < 0.05, **p < 0.01, and ***p < 0.0001.

At the stipulated maturation period (144 h), the metabolic activities of mature biofilms in both atmospheres of oxygen were compared (Figure 2). For EH-315 strain (Figure 2A), the microaerophilic condition resulted in higher metabolic activity of mature biofilms in all media tested when compared to aerobiosis (*p* < 0.0001). The same was observed for the G186A strain with less significance, in the sessile communities formed in the HAM-F12 and RPMI (*p* < 0.01) and BHI and DMEM media (*p* < 0.05) (Figure 2B).

Regarding the condition of microaerophilia, HAM-F12 medium stimulated the higher metabolic activity for the biofilms of both strains when compared to the BHI media (*p* < 0.05 for the EH 315 strain; *p* < 0.01 for the G186A strain), DMEM (*p* < 0.05 for the EH 315 strain; *p* < 0.0001 for the G186A strain), and RPMI (*p* < 0.01 for the EH 315 strain; *p* < 0.0001 for the G186A strain).

### Quantification of Biomass by Crystal Violet Staining

**FIGURE 3:**
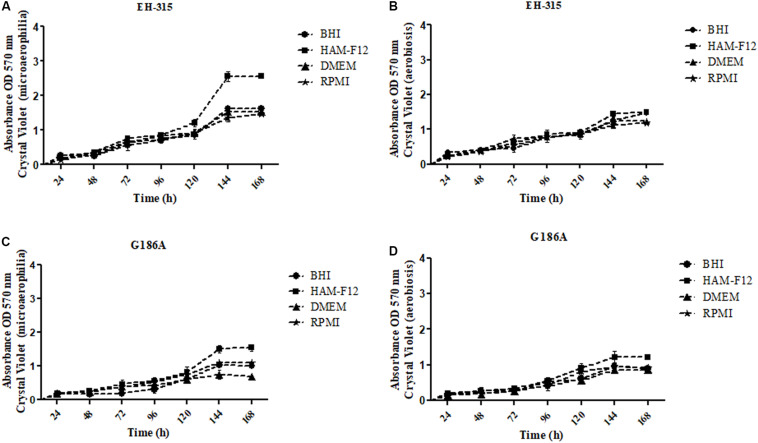
Kinetics of the biomasses formed by different strains of *H. capsulatum* **(A,B)** EH-315 and **(C,D)** G186A in microaerophilia **(A–C)** and aerobiosis **(B–D)** conditions.

The kinetics of the development of biomass in different media and oxygen atmospheres are represented in Figure 3. Both strains EH-315 (Figures 3A,B) and G186A (Figures 3C,D), in all conditions tested (different media and oxygen atmosphere) show similar biomass development up to the period of 120 h. The amount of biomass continued to grow relatively exponentially until 144 h. After this period (144 h), it reaches a plateau corroborating the results of metabolic activities, reinforcing this time as ideal for the maturation of the biofilms of *H. capsulatum.*

**FIGURE 4:**
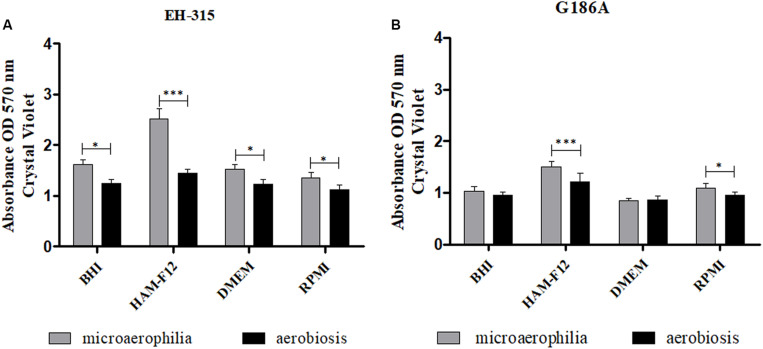
Comparison between the biomasses formed by the strains *H. capsulatum* EH-315 **(A)** and G186A **(B)** in different culture media and under different oxygen atmospheres. *p < 0.05, **p < 0.01, and ***p < 0.0001.

The results of the influence of oxygen atmospheres on the formation of the mass of mature biofilms are shown in Figure 4. The incubation in microaerophilia provided greater biomass formation for both strains in HAM-F12 media (*p* < 0.0001). For the other media, the condition of microaerophilia was slightly higher (*p* < 0.05) or similar to aerobiosis. The results of the quantification of biomass are consistent with those found in the quantification of metabolic activities, proving mainly the efficiency of the HAM-F12 medium in the biofilm formation.

### Quantification of the Extracellular Matrix and Polysaccharide Structures by Safranin Staining

**FIGURE 5:**
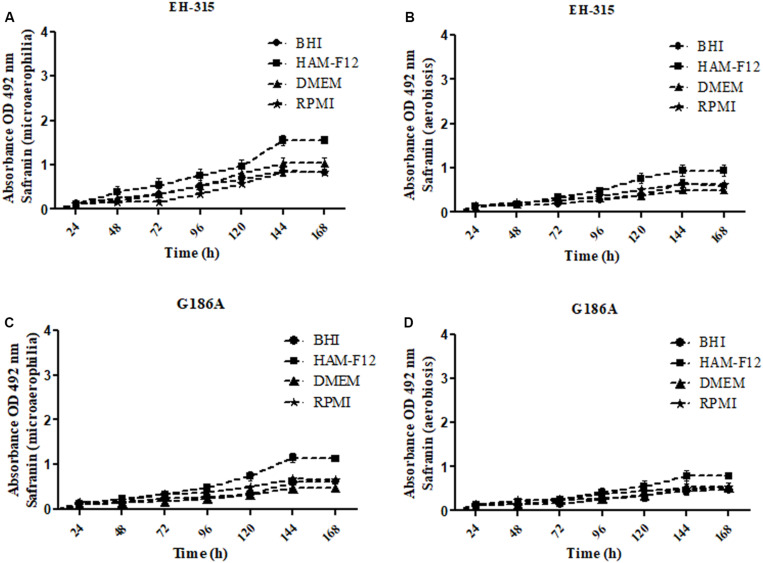
Kinetics of the amounts of extracellular matrix and other external polysaccharides of biofilms formed by different strains of H. capsulatum **(A,B)** EH-315 and **(C,D)** G186A in microaerophilia **(A–C)** and aerobiosis **(B–D)** conditions.

The extracellular matrix and the polysaccharide material produced by the biofilms were quantified using the safranin staining method. Similar patterns were observed in the items above – a slight growth up to 144 h, followed by a stationary period (Figure 5).

**FIGURE 6:**
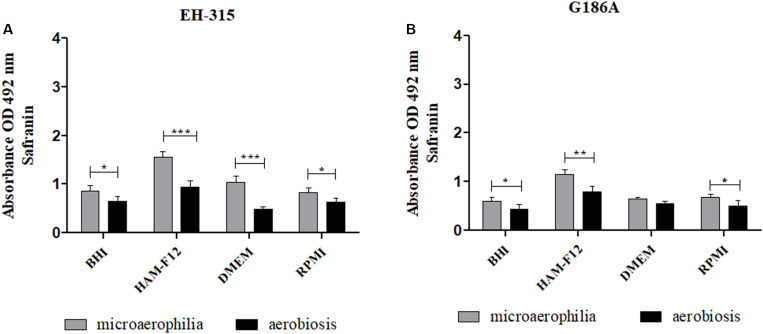
Comparison between the amounts of extracellular matrix and other external polysaccharides formed by the biofilms of *H. capsulatum* EH-315 **(A)** and G186A **(B)** in different culture media and under different oxygen atmospheres. *p < 0.05, **p < 0.01, and ***p < 0.0001.

When verifying the influence of oxygen tension on the development of the matrix and polysaccharide material of mature biofilms, it was observed that both oxygen tensions provided significant extracellular matrix production. However, when compared, the atmosphere with lower oxygen concentration provided better formation of polysaccharide material in relation to the atmosphere with high O_2_ concentration (*p* < 0.05) (Figure 6). Regarding the influence of the culture media, biofilms formed in the HAM-F12 medium produced more extracellular matrices when compared to the others (*p* < 0.01).

### Scanning Electron Microscopy of Biofilms Formed in Different Media

**FIGURE 7:**
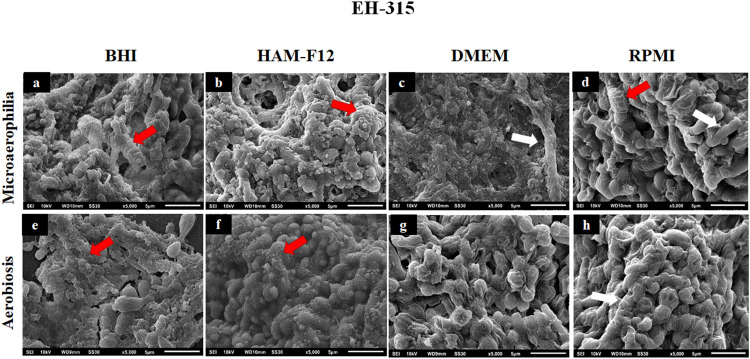
Scanning electron microscopies of the biofilm of *Histoplasma capsulatum* formed by strain EH-315 in different culture media in the conditions of microaerophilia **(a–d)** and conditions aerobiosis **(e–h)**. The red arrows denote the extracellular matrices, while the white arrows denote some hyphae.

**FIGURE 8:**
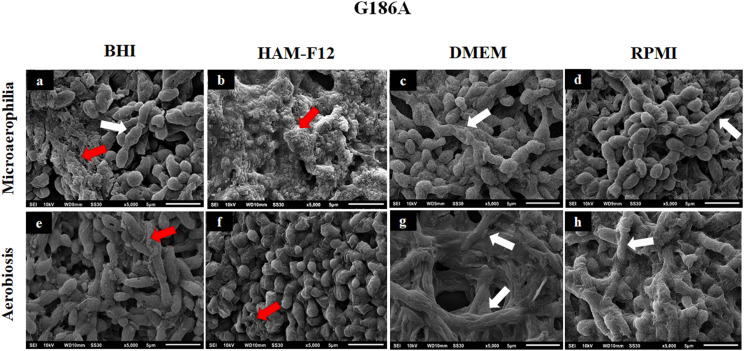
Scanning electron microscopies of the biofilm of *Histoplasma capsulatum* formed by strain G186A in different culture media in the conditions of microaerophilia **(a–d)** and conditions aerobiosis **(e–h)**. The red arrows denote the extracellular matrices, while the white arrows denote some hyphae.

Figures 7, 8 show the SEM images of mature *H. capsulatum* biofilms in different culture media and oxygen atmospheres. The topographies of biofilms formed in the medium HAM-F12 in microaerophilia (**b**) and aerobiosis (**f**) showed yeasts involved in several points by an exuberant amount of extracellular matrix, which is even more evident in the condition of microaerophilia, mainly for the EH 315 strain (Figure 7b). However, in biofilms formed in BHI (**a, e**), DMEM (**c, g**), and RPMI (**d, h**), both different oxygen conditions promoted the formation of a significant amount of extracellular matrix. In addition, significant yeast reversion to hyphae was observed, which requires further investigation.

## Discussion

Despite the efforts of several research groups, the pathogenesis of histoplasmosis still has many gaps, from the mechanisms of interaction with the host, to virulence factors (Adenis et al., 2014b). Recently, one of the biggest challenges related to the treatment of diseases caused by microorganisms, whether they are fungi or not, is biofilm formation. These microorganism communities are considered an important virulence factor (Ramage et al., 2012; Adenis et al., 2014b; Sardi et al., 2015; Costa-Orlandi et al., 2017). Cells from sessile communities can modulate metabolic activity, dormancy, and stress responses, factors that highlight the importance of understanding the properties of the formation of these communities for the investigation of new therapeutic targets (Percival et al., 2012; Pitangui et al., 2012; Mitchell et al., 2015; Costa-Orlandi et al., 2017).

Approaches to fungal biofilms are widely discussed in the literature. Regarding *H. capsulatum*, the first study on the biofilms formed by these fungi was conducted by Pitangui et al. (2012). The authors used BHI medium and techniques such as the XTT reduction assay, fluorescence, confocal and scanning electron microscopies in conditions rich in oxygen. Our results show unprecedented data on the formation of these biofilms using additional techniques and different culture media, in order to verify the influence on their development, as well as the importance of the presence of more or less oxygen, since this fungus is intracellular facultative of alveolar macrophages (Woods, 2003; Garfoot and Rappleye, 2016).

The development of biofilms can be influenced by environmental conditions, nutrients, and pH, which can significantly affect not only the architecture of the biofilm, but also the expression profile of various genes involved during its different stages of formation (Kucharíkova et al., 2011; Tan et al., 2016; Lagree and Mitchell, 2017). Culture media are essential for the growth and development of any microorganism *in vitro*. They are constituted of amino acids, vitamins, inorganic salts, and can differ in number and quantity (Hong et al., 2017; McTaggart et al., 2018). In this work, biofilms were formed in culture media widely used in the cultivation and maintenance of fungi and cell lines. The verification of different nutritional conditions in the formation of these sessile communities is important in order to expand the *in vitro* studies and, mainly, for this phenomenon to be correlated to the pathogenesis of this fungus in future studies.

The culture media tested in this study have a different chemical composition (Supplementary Table S1). BHI medium consists of an infusion of brain-heart, sodium hydrogen phosphate, glucose, peptone, and sodium chloride. These components provide a suitable environment for the growth of fastidious and non-fastidious microorganisms (Rosenow, 1919; Cleare et al., 2020). HAM-F12 medium is widely used for the cultivation and maintenance of mammalian cell cultures (Ham, 1965; Cravchik et al., 1996). In addition to its usual composition (Supplementary Table S1), when supplemented with (18.2 g/L) glucose, (1 g/L) glutamic acid, (6 g/L) HEPES and (8.4 mg/L) L-cysteine, this medium provides a better growth of *H. capsulatum*, since these components are known as growth factors of the fungus (Worsham and Goldman, 1988). Therefore, this medium is used by several authors for the cultivation and maintenance of *H. capsulatum* yeasts (Allendoerfer et al., 1997; Nosanchuk et al., 2003; Guimarães et al., 2011; Cordero et al., 2016; Baltazar et al., 2018; Guimarães et al., 2019; Cleare et al., 2020). The RPMI-1640 medium is considered suitable for growing a variety of mammalian cells (Moore et al., 1967; Trono et al., 2011). In addition, when supplemented with glutamine, 2% glucose, in the absence of sodium bicarbonate and buffered with 3- (N-morpholine) propanesulfonic acid (MOPS) is recommended by the Clinical Laboratory Standard Institute susceptibility testing (CLSI, 2008). DMEM is widely used as a basal culture medium for the growth of various mammalian cell lines and can be supplemented with fetal bovine serum to promote better cell growth (Eagle, 1955; Dulbecco and Freeman, 1959; Burgener and Butler, 2006). Initially, this culture medium was formulated with a low concentration of glucose and pyruvate however, it is often used with a high concentration of glucose and without sodium pyruvate (Dulbecco and Freeman, 1959).

The BHI medium is difficult to compare because it is composed of infusions of brain-heart. This can come from different animals, therefore varying their composition in each batch (Cleare et al., 2020). The other culture media (DMEM, RPMI-1640 and HAM-F12) are defined media, have their balanced composition, in addition to all the known and easy to compare constituents (Supplementary Table S1). However, supplementation with fetal bovine serum in the DMEM medium used in this work, makes the comparison with other media limited. RPMI 1640 is the only one with a glutathione reducing agent, an important antioxidant for cells (Odkhuu et al., 2015). HAM medium has linoleic acid, lipoic acid, thymidine, cupric sulfate, and zinc sulfate as differentiated constituents from other media. Among these constituents, linoleic and lipoic acids are important for the energy metabolism of cells (Casu et al., 2016, 2018; Zhang et al., 2017). Thymidine is important in cell multiplication (Shields et al., 1998). In addition, there is the presence of metallic components, zinc sulfate and cupric sulfate, which are important during the *Histoplasma*-host interaction (Wilson et al., 2012; Gerwien et al., 2018; Cleare et al., 2020). Zinc and copper participate in the catalytic center of numerous enzymes and make important roles in the functionality of several proteins (Van Ho et al., 2002). Several forms of zinc and copper acquisition by some pathogenic fungi have been elucidated, including *S. cerevisiae*, *Aspergillus fumigatus*, *Candida albicans*, *C. neoformans*, *C*ryptococcus *gattii*, *Paracoccidioides* spp., and *H. capsulatum* (Zhao and Eide, 1996; Kim et al., 2008; Nobile et al., 2009; Ganguly et al., 2011; Finkel et al., 2012; Schneider et al., 2012; Parente et al., 2013; Amich and Calera, 2014; Dade et al., 2016). Limiting the access of micronutrients such as copper and zinc within the macrophage inhibits the cell viability of *H. capsulatum* (Dade et al., 2016; Cleare et al., 2020). These data may justify the better growth and development observed in biofilms formed in the HAM-F12 medium, since it contains nutrients that increase the mechanisms of energy metabolism, cell division and the survival of biofilm cells.

With regard to the different oxygen atmospheres used in the present work, they were tested because the oxygen levels are different in the human body (14% in the pulmonary alveoli, 12% in the arterial blood, 5.3% in the venous blood) (Erecińska and Silver, 2001; Sharp and Bernaudin, 2004; Dubois et al., 2016). The microaerophilia formed in the candle jar is a widely used method and is described in the literature as effective for reducing oxygen tension for the growth of some microorganisms *in vitro* (Mandal et al., 2014; Carr et al., 2015). Although the two oxygen tension conditions tested provided a good development of biofilms, in quantitative tests, the condition of microaerophilia was superior to the condition of aerobiosis, particularly in biofilms formed using HAM-F12. A similar profile was observed by Osgood et al. (2015) for the biofilms of *Haemophilus influenzae* formed in the condition of microaerophilia and with neutral pH. *H. capsulatum*, being a systemic pathogen, can develop in different regions of the body and with different oxygen tensions, therefore, verifying the influence of a lower or higher oxygen concentration in the development of biofilms is of paramount importance.

Regarding the filamentation observed in biofilms formed in BHI, RPMI, and DMEM, more studies are needed to unveil this process. One hypothesis may be related to the limitation of essential nutrients in these media, causing stress that stimulates the transition from the yeast to the filamentous phase. The HAM-F12 medium provided the best development of *H. capsulatum* biofilms, presenting little or no visible filament and quantitatively, it was the one that stimulated greater production of biomass, polysaccharide material and more cellular metabolic activity.

Colorimetric methods for quantifying biofilms are established in the literature and are widely used for the study of biofilms (Costa-Orlandi et al., 2017; Wu et al., 2017). The XTT reduction assay quantifies metabolic activity, for this, metabolically active cells reduce the tetrazolium salt (yellow) to formazan salt (orange) (Jiang et al., 2016; Cen et al., 2018). Kinetics studies of biofilm development by XTT assay showed a maturation time of 48 h for *C. neoformans* (Martinez and Casadevall, 2007), 24 h for *Candida* spp. (Chandra et al., 2001; Ramage et al., 2001; Bizerra et al., 2008; Chandra and Mukherjee, 2015; Kean et al., 2018), 72 h for *T. rubrum*, *T. mentagrophytes* (Costa-Orlandi et al., 2014) and *A. fumigatus* (Beauvais and Latgé, 2015) and 144 h for *P. brasiliensis* (Sardi et al., 2015). The maturation time of biofilms in both strains of *H. capsulatum*, formed in this study was 144 h for all culture media and oxygen atmosphere tested. The XTT assay also demonstrated a greater metabolic activity for biofilms formed in the condition of microaerophilia and the HAM-F12 medium showed a greater amount of metabolically active cells. Many authors assume that this assay is an efficient way to quantify biofilms, however it is essential to perform other tests for complete characterization (Marcos-Zambrano et al., 2014; Rosato et al., 2016).

The results obtained in the quantification of the biomass by the crystal violet test, showed a stationary phase in the development of the biomass after 144 h and greater amount of mass in biofilms formed in HAM-F12 medium in the condition of microaerophilia, showing a concordance in what was observed in the XTT assay. Crystal violet is a dye widely used in fungal and bacterial biofilms (Costa-Orlandi et al., 2014; Oliveira et al., 2018; Xu et al., 2019). Basically, it is an assay able to quantify biomass. However, quantification does not make a difference between dead and live cells (Peeters et al., 2008; Xu et al., 2016).

Regarding safranin, the results complement and reaffirm what was observed in the characterization tests described above (XTT and crystal violet). The safranin staining assay is based on the same principle as the crystal violet, but stains only the extracellular matrix and cell polysaccharides (Stiefel et al., 2016; Ommen et al., 2017). Previous studies have shown the efficiency of safranin staining in quantifying biofilms of *Candida*, *Aspergillus*, and dermatophytes species (Seidler et al., 2006, 2008; Costa-Orlandi et al., 2014; Ramos et al., 2017). The micrographs obtained in SEM, confirmed that the nutrients contained in the HAM-F12 medium promoted the formation of a denser and more compact biofilm, in addition to an exuberant amount of extracellular matrix when compared to the other media.

## Conclusion

This is the first study on the influence of nutritional conditions and oxygen concentrations on the biofilm formation by *H. capsulatum*. This study showed that in all tested conditions, it was possible to stimulate the formation of microorganism communities. All tested oxygen media and tensions promoted the development of biofilms, however, those formed in the HAM-F12 medium and in lower O_2_ concentrations were denser and more filled with polysaccharide material. The results of the present work contribute to a better understanding of the biofilm formation by *H. capsulatum* in *in vitro* conditions and open other doors for subsequent studies, involving the influence of specific components of the culture media in the formation of these communities.

## Data Availability Statement

The datasets generated for this study are included in the article.

## Author Contributions

LG, CC-O, NB, and CV drafted the manuscript. LG, CC-O, NB, CV, and RD performed the experiments and analyzed the data. LG, CC-O, and AF-A designed and supervised the project. All authors read and approved the final manuscript.

## Conflict of Interest

The authors declare that the research was conducted in the absence of any commercial or financial relationships that could be construed as a potential conflict of interest.

## Footnotes

1 www.histoplas-mex.unam.mx
