# Supplementary material for: Biofilm Formation by Histoplasma capsulatum in Different Culture Media and Oxygen Atmospheres
Source: Front Microbiol. 2020 Jul 10;11:1455. doi: 10.3389/fmicb.2020.01455 (PMC7365857; doi:10.3389/fmicb.2020.01455)
Supplement: Supplementary file 1 [file Table_1.docx]

| Table S1. Composition of BHI, HAM-F12, DMEM and RPMI. | | | | | |  |  |  | | |  |
| --- | --- | --- | --- | --- | --- | --- | --- | --- | --- | --- | --- |
|  |  | **Culture medium** | |  | | | | |  |  |  |
| Components | **BHI** | **HAM-F12** | **DMEM (High glucose)** | | **RPMI** | | | | |  |  |
| Amino acids | _ | L-Alanine  L-Arginine - HCl  L-Asparigine – H_2_O  L-Aspartic Acid  L-Cysteine – HCl -H_2_O  L-Glutamic Acid  L-Glutamine  Glycine  L-Histidine – hydrochoride– H_2_O  L-Isoleucine  L-Leucine  L-Lysine - HCl  L-Methionine  L-Phenylalanine  L-Proline  L-Serine  L-Threonine  L-Tryptofan  L-Tyrosine – 2Na – 2H_2_O  L-Valine | Glycine  L-Arginine hydrochloride  L-Cystine – 2HCl  L-Glutamine  L-Histidine hydrochloride – H_2_O  L-Isoleucine  L-Leucine  L-Lysine hydrochloride  L-Methionine  L-Phenylalanine  L-Serine  L-Threonine  L-Tryptophan  L-Tyrosine disodium salt dihydrate  L-Valine | | Glycine  L-Arginine  L-Asparigine  L-AspArtic acid  L-Cystine 2HCl  L-Glutamic Acid  L-Glutamine  L-Histidine  L-Hydroxyproline  L-Isoleucine  L-Leucine  L-Lysine hydrochloride  L-Methionine  L-Phenylalanine  L-Proline  L-Serine  L-Threonine  L-Tryptophan  L-Tyrosine disodium salt dihydrate  L-Valine | | | | |  |  |
| Vitamins | _ | Biotin  Choline Chloride  Folic Acid  i- Inositol  Niacinamide  D- Calcium pantothenate  Pyridoxine - HCl  Riboflavin  Thiamine - HCl  Vitamin B_12_ | Choline chloride  D-Calcium pantothenate  Folic Acid  Niacinamide  Pyridoxine hydrochloride  Riboflavin  Thiamine hydrochloride  i-Inositol | | Biotin  Choline chloride  D-Calcium pantothenate  Folic Acid  Niacinamide  Para-Aminobenzoic Acid  Pyridoxine hydrochloride  Riboflavin  Thiamine hydrochloride  Vitamin B12  i-Inositol | | | | |  |  |
| Inorganic salts | Sodium chloride  Disodium phosphate | Calcium Chloride (anhyd.)  Cupric Sulfate – 5H_2_O  Ferrous Sulfate – 7H_2_O  Magnesium Chloride (anhydrous)  Potassium Chloride  Sodium Bicarbonate  Sodium Chloride  Sodium Phosphate Dibasic (anhydrous)  Zinc Sulfate – 7H_2O_ | Calcium Chloride (anhyd.)  Ferric Nitrate - 3”9H_2_O  Magnesium Sulfate (anhyd.)  Potassium Chloride  Sodium Bicarbonate  Sodium Chloride  Sodium Phosphate monobasic – H_2_O | | Calcium nitrate (4H_2_0)  Magnesium Sulfate (anhyd.)  Potassium Chloride  Sodium Bicarbonate  Sodium Chloride  Sodium Phosphate dibasic anhydrous | | | | |  |  |
| Others components | Calf brain  Beef heart  Proteose peptone  D-Glucose | D-Glucose  Hypoxanthine Na  Linoleic Acid  Lipoic Acid  Phenol Red  Putrescine - 2HCl  Sodium Pyruvate  Thymidine | D-glucose (Dextrose)  Phenol Red | | D-Glucose (Dextrose)  Glutathione  Phenol Red | | | | |  |  |
| Additional supplementation | D-glucose  L-Cysteine | D-glucose  L-Glutamic Acid  Hepes  L-Cysteine | Heat-inactivated fetal calf serum  Sodium bicarbonate | | D-glucose  MOPS | | | | |  |  |
